# DP2, a Carbohydrate Derivative, Enhances In Vitro Osteoblast Mineralisation

**DOI:** 10.3390/ph16111512

**Published:** 2023-10-24

**Authors:** Nissrine Ballout, Agnès Boullier, Walaa Darwiche, Katia Ait-Mohand, Eric Trécherel, Théo Gallégo, Cathy Gomila, Linda Yaker, Isabelle Gennero, José Kovensky, Jérôme Ausseil, Sylvestre Toumieux

**Affiliations:** 1 Société d’Accélération du Transfert de Technologie-Nord, 59800 Lille, France; nissrine.ballout@inserm.fr (N.B.);; 2 Institut Toulousain des Maladies Infectieuses et Inflammatoires, INSERM UMR1291, CNRS UMR5051, University of Toulouse III, 31024 Toulouse, France; 3 Service de Biochimie, Institut Fédératif de Biologie, CHU Toulouse, 31024 Toulouse, France; 4 Mécanismes Physiopathologiques et Conséquences des Calcifications Cardiovasculaires, UR7517, Centre Universitaire de Recherche en Santé, CURS-UPJV, University of Picardy Jules Verne, 80054 Amiens, Francetrecherel.eric@chu-amiens.fr (E.T.);; 5 Laboratory of Biochemistry, CHU Amiens-Picardie, 80054 Amiens, France; 6 Laboratoire de Glycochimie et des Agroressources d’Amiens, UR 7378, CNRS, Université de Picardie Jules Verne, 80039 Amiens, France

**Keywords:** DP2, bone morphogenetic proteins, osteoblast, bone regeneration

## Abstract

Bone fracture healing is a complex biological process involving four phases coordinated over time: hematoma formation, granulation tissue formation, bony callus formation, and bone remodelling. Bone fractures represent a significant health problem, particularly among the elderly population and patients with comorbidities. Therapeutic strategies proposed to treat such fractures include the use of autografts, allografts, and tissue engineering strategies. It has been shown that bone morphogenetic protein 2 (BMP-2) has a therapeutic potential to enhance fracture healing. Despite the clinical efficacy of BMP-2 in osteoinduction and bone repair, adverse side effects and complications have been reported. Therefore, in this in vitro study, we propose the use of a disaccharide compound (DP2) to improve the mineralisation process. We first evaluated the effect of DP2 on primary human osteoblasts (HOb), and then investigated the mechanisms involved. Our findings showed that (i) DP2 improved osteoblast differentiation by inducing alkaline phosphatase activity, osteopontin, and osteocalcin expression; (ii) DP2 induced earlier in vitro mineralisation in HOb cells compared to BMP-2 mainly by earlier activation of Runx2; and (iii) DP2 is internalized in HOb cells and activates the protein kinase C signalling pathway. Consequently, DP2 is a potential therapeutical candidate molecule for bone fracture repair.

## 1. Introduction

Bone is a highly vascularized and resilient organ, with innate abilities to repair, regenerate, and restore function after fracture. In 5 to 10% of the cases, fractured bones experience impaired healing (malunion or non-union) related to mechanical and biological environmental factors such as ageing, smoking, alcoholism, and medical conditions such as type I diabetes, anaemia, tumours, malformations, and osteoporosis [1]. In 2019, almost 4.3 million bone fractures occurred in European countries [2,3]. This includes 484,000 osteoporotic fractures in people over 50 years old in France [2,3].

Fracture healing is a complex process that starts with the formation of a hematoma following vascular disruption, and the formation of a clot at the site of the lesion. A non-infectious inflammatory response follows coagulation. Then, neutrophils and macrophages enter the fracture site, phagocytose the debris, and initiate the repair cascade by promoting an angiogenic response [4]. A few days after the fracture, osteoclasts begin to shed the necrotic tissue. Revascularization of damaged tissue leads to the recruitment of osteochondroprogenitor mesenchymal cells [5]. After two to three weeks, the hematoma is replaced by an extracellular matrix of fibroblasts and chondroblasts derived from recruited progenitor cells, thus developing the soft callus [6]. During this process, the differentiation of progenitor cells into osteoblasts marks the beginning of intramembrane bone growth. Over the course of 3 to 4 months, endochondral ossification is initiated inside the fracture to allow for the formation of woven bone, allowing the soft callus to slowly transform into a hard callus. After the completion of the hard callus, during the following months or years, a second phase of resorption is initiated in order to remodel the hard callus into the lamellar bone, which allows for the restoration of bone properties [7].

Fracture healing impairments are a major health problem and require additional medical interventions, which results in significant costs to the healthcare system. Therapeutic strategies proposed to treat such fractures include the use of autografts, allografts, and tissue engineering strategies [8,9,10]. However, while autografts are often associated with donor site morbidity and a lack of appropriate donor sites, allografts are associated with immunogenic rejection and a risk of infection [8]. Therefore, research has focused on new strategies such as bone tissue engineering which involves the use of cell therapy, scaffolds, growth factors (GF), or osteoinductive molecules. Specifically, GFs such as fibroblast growth factors, bone morphogenetic proteins (BMPs), insulin-like growth factor, platelet-derived growth factor, transforming growth factor-beta, and vascular endothelial growth factor have been used in bone tissue engineering either directly or delivered via a scaffold [11]. Indeed, BMPs 2, 4, 6, 7, and 9 have bone induction capacities [12,13,14] and were extensively used in bone regeneration [15]. BMP-2 is FDA-approved for orthopaedic and craniofacial surgery under the names of Inductos^®^ and Infuse^®^ Bone Graft (Medtronic, Dublin, Ireland) [16]. Despite the clinical efficacy of BMP-2 in osteoinduction and bone repair, adverse side effects and complications were reported, such as extreme inflammation and oedema, and uncontrolled ectopic bone formation [17,18,19,20,21]. In addition, hBMP-2 is derived from Chinese hamster ovary cells via genetic recombination methods, which generate a high cost of manufacturing and a restriction in widespread clinical use [22]. Moreover, the reconstituted BMP-2 is introduced on an absorbable collagen scaffold (ACS), which retains a small percentage of BMP-2 at the implant site resulting in irregular bone growth and an increased risk of adverse side effects. Due to these side effects and the lack of good scaffold manufacturing practices, the production of Inductos^®^ and Infuse^®^ was suspended in 2016 in Europe.

Previous promising results in our laboratory showed that DP2, a well-tolerated saccharide compound obtained after a convergent synthesis, starting with glucose [23], had the ability to enhance inorganic induced calcification in pre-osteoblastic murine cells (MC3T3). Therefore, in this present study, we evaluated the in vitro effect of DP2 on the mineralisation of primary human osteoblastic cells (HOb), and studied the mechanisms involved in this process.

## 2. Results

### 2.1. DP2 Does Not Affect the Viability of HOb Cells

**Figure 1:**
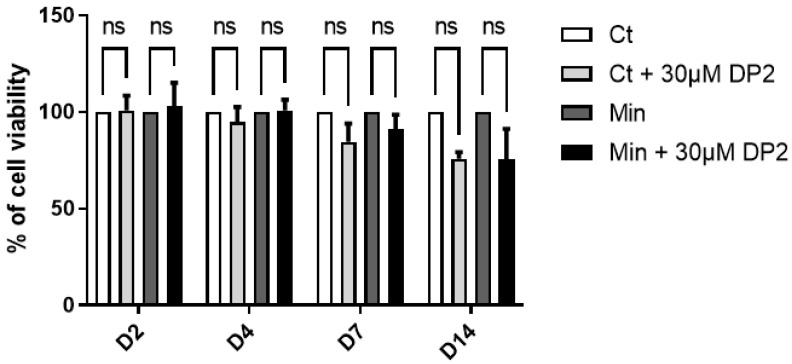
**Effect of DP2 on HOb cell viability.** HOb cells were incubated with either the culture medium (Ct), Ct + 30 μM DP2, mineralising medium (Min), or Min + 30 μM DP2 for 2, 4, 7, and 14 days (D). The cell viability was then assessed using the MTT test. The cell viability of untreated cells (Ct) was considered as 100%. Data are expressed as the mean ± SEM of three independent experiments performed in triplicate (*n* = 3). ns: non-significant.

An MTT test was performed to rule out any cytotoxic effects of DP2 on the cells. In brief, cells were treated with either the culture medium (Ct), Ct + 30 μM DP2, mineralising medium (Min), or Min + 30 μM DP2 for 2, 4, 7, and 14 days (D). No significant difference was observed between the different treatment conditions and incubation times (Figure 1). On day 14, we observed a decrease in cell viability, which is due to osteoblast apoptosis in the process of calcification in vitro. These results suggest that 30 μM DP2 exhibits no cytotoxicity in HOb cells.

### 2.2. DP2 Promotes Calcification and Induces Alkaline Phosphatase (ALP) Activity in HOb Cells

**Figure 2:**
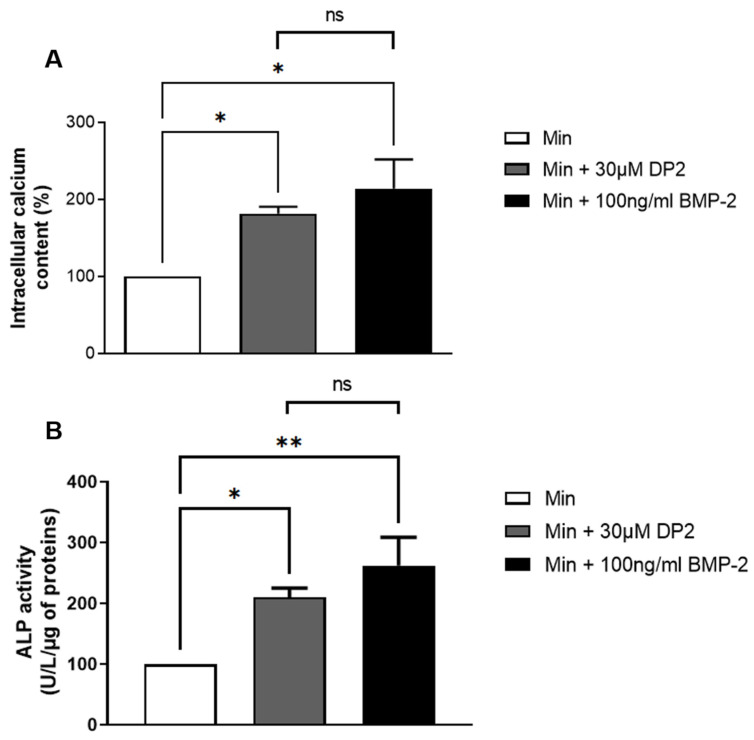
**Effect of DP2 on HOb cell calcification and alkaline phosphatase (ALP) activity**. (**A**) HOb cells were incubated with either the mineralised medium (Min), Min + 30 μM DP2, or Min + 100 ng/mL BMP 2 for 14 days. The intracellular calcium content was quantified using the OCP colorimetric method. The intracellular calcium content of Min-treated cells (Min) was considered as 100% (*n* = 4). (**B**) HOb cells were incubated with either Min, Min + 30 μM DP2, or Min + 100 ng/mL BMP-2 for 7 days. The enzymatic activity of Min-treated cells was considered as 100% (*n* = 5). Data are expressed as the mean ± SEM. The *p*-value was determined using a one-way ANOVA, followed by Tukey’s multiple comparisons test (* *p* < 0.05, ** *p* < 0.01); ns: non-significant.

During bone mineralisation, osteoblasts participate to the release of calcium phosphate crystals [24]. The pro-calcifying activity of DP2 was determined by treating HOb cells in a mineralising medium with 30 µM DP2 for 14 days, and compared to HOb cells treated with 100 ng/mL BMP-2 used as a positive control. The concentration of DP2 was determined based on a pilot study that showed that mineralisation is detected starting at 30 µM DP2. As for BMP-2, 100 ng/mL was the concentration used in several similar studies [25,26,27]. Our results showed that both 30 μM DP2 and 100 ng/mL BMP-2 increase the mineralisation of HOb cells compared to the mineralising medium (Min) alone (181.57 ± 9.16% and 214.16 ± 37.94% respectively, Figure 2A). This indicates that 30 μM DP2 has a pro-calcifying similar activity to 100 ng/mL BMP-2. The ALP enzyme plays an essential role in bone formation. ALP regulates pyrophosphate levels and reduces pyrophosphate levels to promote calcification [28]. During osteoblast differentiation, the expression of ALP by osteoblasts increases gradually over time. We therefore measured ALP activity seven days post-treatment and showed a significant increase in the presence of 30 µM DP2 (210.49 ± 14.98%, *p* < 0.05) or 100 ng/mL BMP-2 (262.57 ± 46.21%, *p* < 0.01) compared to the mineralised medium (Min) alone (100%; Figure 2B).

### 2.3. DP2 Induces Calcification Marker Expression Earlier Than BMP-2

**Figure 3:**
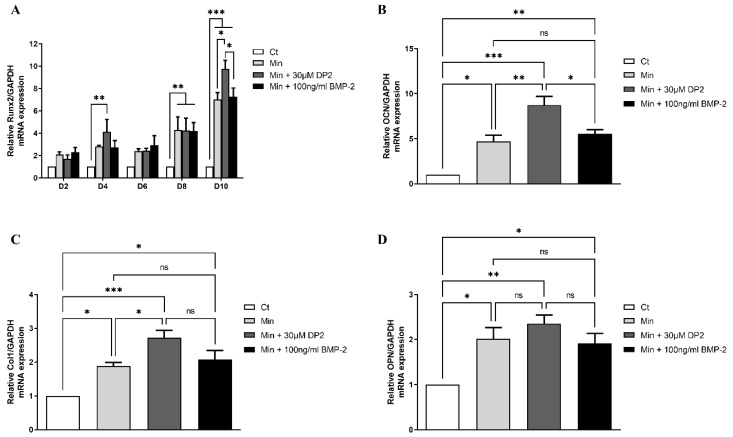
**Effect of DP2 on HOb cell osteoblastic differentiation**. (**A**) HOb cells were incubated with either a medium alone (Ct), mineralised medium (Min), Min + 30 μM DP2, or Min + 100 ng/mL BMP-2 for 2 days (D2), 4 days (D4), 6 days (D6), 8 days (D8), or 10 days (D10). Data are expressed as the mean ± SEM (*n* = 3). The *p*-value was determined using Two-way ANOVA followed by Tukey’s multiple comparisons test (* *p* < 0.05, ** *p* < 0.01, *** *p* < 0.001). (**B**–**D**) Gene expression of (**B**) osteopontin (OCN), (**C**) type I collagen (Col1), and (**D**) osteopontin (OPN) was measured 10 days after treatment with either Ct, Min, Min + 30 μM DP2, or Min + 100 ng/mL BMP-2. Data are expressed as the mean ± SEM (*n* = 3). The *p*-value was determined using a one-way ANOVA followed by Tukey’s multiple comparisons test (* *p* < 0.05, ** *p* < 0.01, *** *p* < 0.001); ns: non-significant.

Several signalling pathways are involved in osteoblastic differentiation and in the induction of mineralisation, such as the MAPK (mitogen-activated protein kinases) pathway and the Wnt/β-catenin pathway. These pathways induce the activation of transcription factors involved in osteoblastic differentiation, such as Runx2, Osterix, and Dlx5 [29]. In addition to transcription factors, progressive osteoblast differentiation is characterized by the expression of osteoblastic genes such as collagen 1 (Col1), osteocalcin (OCN), and osteopontin (OPN) [29]. Thus, the gene expression of Runx2, Col1, OCN, and OPN was measured by profiling the mRNA expression in HOb cells treated or not with DP2 and compared to HOb cells treated with 100 ng/mL BMP-2. Our results showed a significant increase in Runx2 gene expression 4 days after DP2 treatment (4.115 ± 1.110, *p* < 0.01) in comparison to the control group (Ct). Interestingly, a peak of expression was observed at day 10 in the DP2 group (9.752 ± 0.778) in comparison to the Min (7.024 ± 0.613, *p* < 0.05) and BMP-2 (7.276 ± 0.768, *p* < 0.05) groups, indicating that DP2 induces calcification earlier than BMP-2 (Figure 3A).The significant increase in DP2 gene expression of the main markers during osteoblast differentiation such as osteocalcin (OCN, Figure 3B), collagen I (Col1, Figure 3C), and osteopontin (OPN, Figure 3D) at day 10 post-treatment is consistent with these results. These findings suggest that DP2 induced osteogenic differentiation.

### 2.4. DP2 Is Internalized in HOb Cells

**Figure 4:**
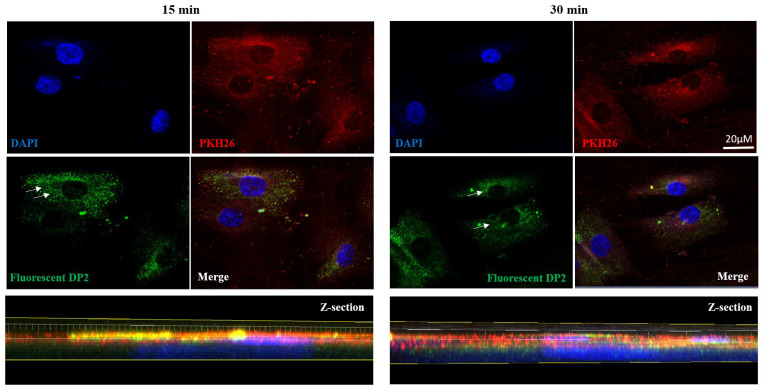
**DP2 internalization in HOb cells.** HOb cells were incubated with the mineralised medium (Min) + 30 μM of fluorescent DP2, and the internalisation was evaluated via confocal microscopy. High-magnification images and Z-sections showing nucleus labelling in blue (DAPI), membrane labelling in red (PKH26), and fluorescent DP2 in green 15 and 30 min after treatment. White arrows show fluorescent DP2 in the cytoplasm. Scale bar: 20 µm.

In order to determine whether DP2 can penetrate HOb cells, we designed a fluorescent DP2 in which a fluorescein probe was linked to DP2 with a peptide bond [30]. HOb cells were then treated with 30 µM of fluorescent DP2 for several incubation periods (15 and 30 min). Double-nuclear (DAPI) and membrane (PKH26) labelling was performed, and the fluorescence was visualized using a confocal microscope. The results presented in Figure 4 show that 15 and 30 min after treatment, fluorescent DP2 was detectable in the cytoplasm (white arrows). Labelling analysis on z-sections confirmed the presence of DP2 in the cytoplasm 30 min post-treatment. These results suggest that DP2 is internalised in osteoblast cells.

### 2.5. DP2-Induced Calcification Could Be Mediated at Least in Part by PKC Signalling

**Figure 5:**
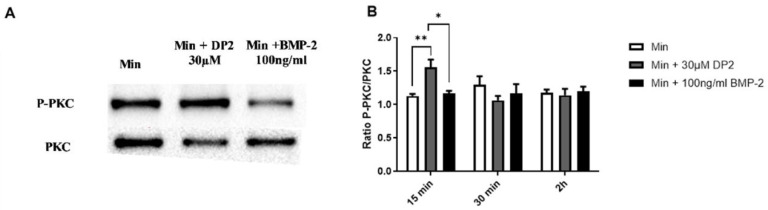
**Activation of the PKC signalling pathway after DP2 treatment.** HOb cells were incubated with either mineralised medium (Min) alone, Min + 30 μM DP2, or Min + 100 ng/mL BMP-2. The protein expression of the phosphorylated and total PKC (**A**) was evaluated by western blotting. Histograms (**B**) represent the ratio between the total and the phosphorylated protein. Data are expressed as the mean ± SEM of three independent experiments performed in quadruplicate (*n* = 4). The *p*-value was determined using a one-way ANOVA followed by Tukey’s multiple comparisons test (* *p* < 0.05, ** *p* < 0.01).

As our results suggest an early internalisation of DP2 in HOb cells, we aimed to determine the molecular mechanisms and the signalling pathways involved in the calcification induced by DP2. The expression of several canonical phosphorylated and total proteins involved in cell differentiation and/or cell migration, such as PKC, Akt, CREB, p38, and ERK 1/2, was then quantified using Western blotting in the presence of DP2 compared to a 100 ng/mL BMP-2 treatment. A treatment kinetic study showed no significant difference in the protein expression level of ERK ½, Akt, CREB, and p38 between the different treatment conditions. However, a significant increase in the protein expression level of phosphorylated PKC was observed 15 min after treatment with Min + 30 μM DP2 (1.55 ± 0.12) compared to the mineralising medium alone (Min: 1.13 ± 0.03) or Min + 100 ng/mL BMP-2 (1.17 ± 0.04) (Figure 5A,B). No difference was observed after 30 min and 2 h of treatment. These results suggest that the effect of DP2 on osteoblastic differentiation could be mediated at least in part by the activation of the PKC signalling pathway. Figure 5B shows that the osteogenic effect of BMP-2 is not mediated by PKC, indicating that DP2 and BMP-2 regulate two different signalling pathways.

### 2.6. Signalling Pathways Implicated in DP2-Induced Mineralisation

**Figure 6:**
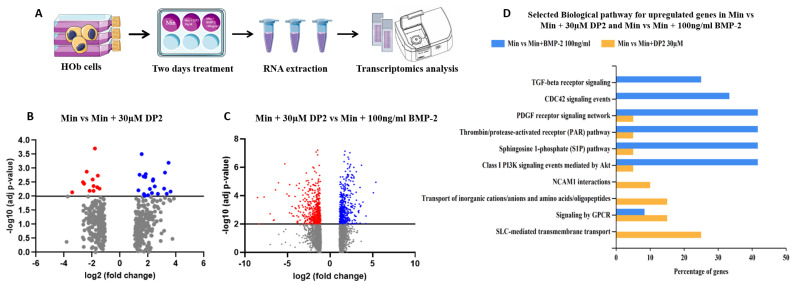
**Effect of DP2 on gene expression in HOb cells.** (**A**) Cell sample preparation: HOb cells were incubated with either the mineralised medium (Min), Min + 30 μM DP2, or Min + 100 ng/mL BMP-2 for 2 days. (**B**) Volcano plot showing the genes (blue) up- or (red) downregulated after treatment with Min + 30 µM DP2 compared to treatment with Min. (**C**) Volcano plot showing the genes up- or downregulated after treatment with Min + 30 µM DP2 compared to treatment with Min + 100 ng/mL BMP-2. (**D**) Gene ontology (GO) enrichment for the upregulated genes in HOb cells treated with Min + 30 µM DP2 compared to treatment with Min + 100 ng/mL BMP-2.

To determine whether DP2 acts according to a different mechanism from BMP-2, the change in gene expression in the presence of DP2 was analysed in a transcriptomic study. This study was performed by comparing the expression of mRNA from HOb cells treated with either Min, Min + 30 µM DP2, or Min + 100 ng/mL BMP-2 using RNA-seq, (Figure 6A). As shown in Figure 6B, the expression of several groups of genes was modified in the presence of 30 µM DP2 compared to the mineralising medium alone (Min). Thirty-two genes showing a significant change in their expression level in the presence of DP2 (*p* < 0.01 compared to Min) were identified. Among them, genes involved in the regulation of ion channels, the expression of G-protein-coupled receptors, and enzymes of the cytochrome P450 family were upregulated.

Interestingly, this transcriptomic study also highlighted significant changes (*p* < 0.01) in the gene expression of 1153 genes between treatment with 30 µM DP2 or 100 ng/mL BMP-2 (Figure 6C). For instance, genes involved in inorganic cation transport and GPCR-mediated signalling were significantly upregulated in the presence of 30 µM DP2. Therefore, DP2 seems to modulate the expression of extracellular proteins as well as membrane proteins. Furthermore, the results of the Gene Ontology (GO) enrichment analysis clearly show that the gene modifications induced by DP2 are different from those induced by BMP-2 (Figure 6D). Biological pathways such as NCAM1 interactions and SLC-mediated transmembrane transport were only upregulated after treatment with 30 µM DP2. Transcriptomic results confirmed that the osteogenic effect of DP2 and BMP-2 is mediated via different pathways.

## 3. Discussion

Impaired fracture healing is a major health problem. Severe bone lesions cause hundreds of millions of surgical procedures worldwide each year. The standard treatment for bone defects is autograft. This graft has the advantage of being osteogenic, osteoinductive, and osteoconductive. However, this technique has significant limitations, including painful and limited access to the graft site, large volumes to be harvested by the surgeon, and harvest site morbidity. Therefore, recent research has focused on new strategies for bone regeneration, such as the use of BMP-2. Despite the clinical efficacy of BMP-2, adverse clinical events such as extreme inflammation and oedema, as well as poor-quality bone regeneration, and uncontrolled ectopic bone formation were observed. In this study, we evaluated the effect of a disaccharide, DP2, on HOb cell calcification, and investigated the molecular mechanisms involved in this process in vitro.

Alongside increased mineralisation, our results showed an increase in ALP activity after treatment with DP2 or BMP-2. ALP is an enzyme that hydrolyses organic phosphate and pyrophosphate and induces bone mineralisation [31]. It is considered an early marker of osteoblastic differentiation and is known to increase the following treatment with BMP-2 [32]. Therefore, our results suggest that DP2 promotes osteoblastic differentiation, in vitro, in a similar manner to BMP-2.

Runx2, a major transcription factor involved in bone formation, is required for the activation of osteoblast differentiation and the expression of the bone formation-related genes ALP, osteocalcin (OCN), and osteopontin (OPN). Interestingly, our results showed that DP2 increases the expression of Runx2 earlier than BMP-2. These findings confirm that by increasing the expression of Runx2, DP2 has a pro-calcifying effect, and suggests that DP2 acts earlier than BMP-2 on Runx2 activation. In addition, Runx2 is a downstream molecule of the mitogen-activated protein kinase (MAPK) signalling pathway activated during osteoblast differentiation [33]. MAPKs are a family of serine/threonine kinases that play significant roles in different cellular processes, such as proliferation, differentiation, and inflammation. It has been reported that the activation of p38 MAPK and ERK 1/2 signalling pathway may up-regulate bone-formation-related gene expression and promote osteoblastic differentiation and mineralisation. In osteoblasts, PKC is an important component in signal transduction pathways, inducing cell proliferation and differentiation [34]. Our results showed that after a 15 min treatment with DP2, PKC was activated, unlike with BMP-2 treatment. In contrast, the protein expression levels of ERK, Akt, CREB, and p38 were not increased. Increased PKC expression is correlated with increased levels of OCN, bone sialoprotein, and ALP [35]. PKC isoforms are expressed during osteogenic differentiation and play a role in the signalling of several osteogenic regulators [36]. However, the osteogenic activity of BMP-2 is repressed by PKC activation by an increase in Msx2 stability [37]. Together, our results demonstrate that although BMP-2 and DP2 induce the activation of Runx2, they do not involve the same signalling cascade. Moreover, the fluorescence results with the labelled DP2 suggest that DP2 can be internalised. In addition, the transcriptomic study suggests that DP2 may act on the cell membrane or with membrane receptors. For instance, genes involved in inorganic cation transport and GPCR-mediated signalling were significantly upregulated in the presence of DP2. Biological pathways, such as NCAM1 interactions, are upregulated after treatment with DP2. NCAM1 was shown to be involved in the osteoblastic differentiation of MC3T3-E1 cells [38]. These interesting results led us to hypothesise that DP2 has a dual mechanism of action to trigger osteoblast differentiation: (i) DP2 signals through the PKC signalling pathway, which regulates the expression of the osteogenic transcription factor Runx2 and induces the transcription of OCN, ColA1, and OPN afterward [39]; and later, (ii) DP2 signals through endocytosis and signal transduction to induce the transcription of Runx2.

## 4. Materials and Methods

### 4.1. DP2 Disaccharide Synthesis

**Figure 7:**
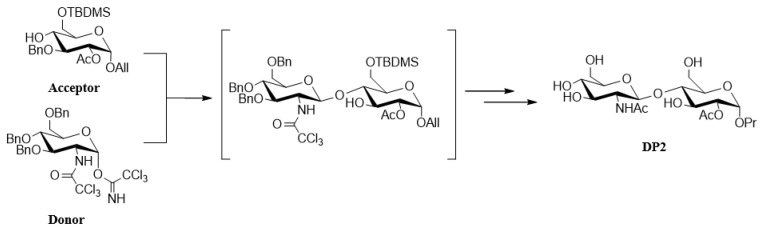
**Synthesis and structure of DP2.** The acceptor glucoside was obtained in eight steps, with total control of the alpha stereochemistry for the aglycone moiety. The alpha trichloroacetimidate donor glucoside was straightforwardly obtained in a three-step sequence [23].

The DP2 disaccharide was obtained after a convergent synthesis starting with glucose [23]. It required the glycosylation of the acceptor glucoside with a glucoside donor. The key glycosylation step reaction is completely stereoselective to afford the disaccharide as a single diastereoisomer leading to the DP2 carbohydrate after deprotection steps (Figure 7).

### 4.2. Fluorescent DP2 Synthesis

**Figure 8:**
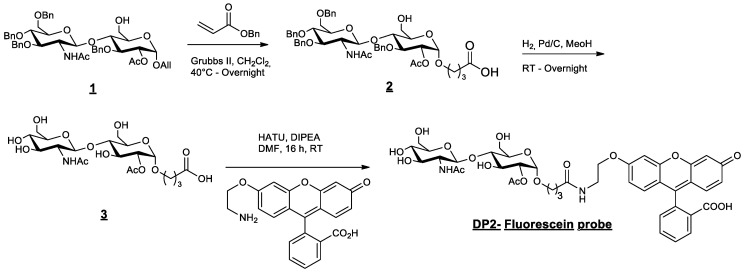
Synthesis and structure of fluorescent DP2.

Substrate **1** (Figure 8) is an intermediate of the synthesis of the acceptor [23]. Compound **1** (1 g, 1.21 mmol) and benzyl acrylate (1.400 g, 7.13 equiv., 8.63 mmol) were dissolved in dichloromethane (100 mL). The solution was degassed with nitrogen. The Grubbs II catalyst (100 mg, 9.6 mol%, 0.11 mmol) was added to the mixture under nitrogen. The reaction was stirred under reflux, left overnight, and concentrated to dryness under reduced pressure. The crude product was directly purified by chromatography over silica gel (CH_2_Cl_2_/Acetone 1:0 to 1:1) to afford compound **2** (714 mg, 61%).

Compound **2** (869 mg, 0.90 mmol) was dissolved in methanol (75 mL). The solution was degassed with nitrogen, and palladium on carbon 10 wt% (290 mg, 0.3 equiv.) was added. Hydrogen was bubbled through the reaction mixture. After stirring the mixture for 24 h at RT, it was filtered through a celite pad and washed with methanol. The filtrate was concentrated under vacuum and co-evaporated with acetonitrile to afford the crude product **3**. The greyish powder (493 mg crude, quant.) was engaged in the next step without any further purification.

Compound **3** (150 mg, 0.293 mmol, 1.1 equiv.) and the fluorescein derivative [30] (110 mg, 0.267 mmol, 1 equiv.) were added to a solution of *N,N*-dimethylformamide (5 mL). *N,N*-diisopropylethylamine (0.26 mL, 1.5 mmol, 5.5 equiv.) was added to the suspension, which changed the colour to orange. The reaction was stirred for 18 h at RT and concentrated under reduced pressure. Traces of DMF were co-evaporated with toluene and acetonitrile. The crude product was successively purified via chromatography over silica gel (CH_2_Cl_2_/MeOH -9:1 to 8:2) and preparative TLC to produce the expected pure DP2–fluorescein probe (49 mg, 21%).

^1^H NMR (400 MHz, CD_3_OD): δ 8.00 (d, J = 7.6 Hz, 1H), 7.74 (m, 2H), 7.20 (d, J = 7.6 Hz, 1H), 6.88 (d, J = 1.9 Hz, 1H), 6.75–6.63 (m, 3H), 6.60–6.50 (m, 2H), 4.91 (d, J = 3.9 Hz, 1H), 4.61 (dd, J = 3.6, 10.0 Hz, 1H), 4.50 (d, J = 8.4 Hz, 1H), 4.10 (t, J = 5.4 Hz, 2H), 3.98–3.88 (m, 2H), 3.78–3.29 (m, 13H), 2.31 (t, J = 7.4 Hz, 2H), 2.05 (s, 3H), 2.00 (s, 3H), 1.87 (t, J = 6.9 Hz, 2H).

^13^C NMR (100 MHz, CD_3_OD): δ 175.8, 173. 8, 172.3, 171.5, 171.5, 162.1, 161.4, 154.2, 154.0, 153.9, 136.6, 131.1, 130.1, 128.1, 125.8, 125.3, 113.7, 113.1, 112.8, 111.2, 103.6, 103.2, 102.7, 97.0, 81.5, 78.1, 75.7, 74.3, 72.0, 71.8, 71.0, 68.3, 67.9, 62.6, 61.6, 57.3, 39.9, 33.5, 26.7, 23.1, 20.8.

### 4.3. Cell Culture

Primary human osteoblast (HOb) cells isolated from human femur trabecular bone tissue were purchased from Promocell (Heidelberg, Germany). Cells were kept at 37 °C in a humidified atmosphere (90% humidity) of 5% CO_2_. HOb cells were cultured in a proliferation medium (human osteoblast proliferation medium, PromoCell, Heidelberg, Germany). To induce mineralisation, HOb cells were treated with an “osteoblast mineralisation medium” (Min) (Promocell, Heidelberg, Germany). The molecules DP2 at 30 µM or BMP-2 at 100 ng/mL (Biotechne, Minneapolis, MN, USA) were then added. A pilot study was conducted using different concentrations, from 5 to 50 µg of DP2 to treat HOb cells; the results showed that mineralisation was detected starting at 30 µM DP2. As for BMP-2, 100 ng/mL was the concentration used in several similar studies [26,36,37].

### 4.4. Cell Viability Assay

Cell viability was determined using the MTT test (3-(4,5-dimethyl-2-thiazolyl)-2,5-diphenyl-2H-tetrazolium bromide, SIGMA, Saint-Louis, MO, USA). In brief, HOb cells were plated in 48-well plates (10,000 cells per well) and treated two days later with 30 μM DP2. The cell viability assay was prepared on days (D) 2, D4, D7, and D14 post-treatment by adding 5 mg/mL MTT to each well. The plates were then maintained at 37 °C with 5% CO_2_ for 1 h. The optical density of formazan was spectrophotometrically measured at 560 nm. The cytotoxicity, expressed as a percentage, was evaluated by comparing the cell viability between the control (untreated cells) and experimental groups (treated cells).

### 4.5. Calcification Assay

The intracellular calcium content was assessed using the o-cresolphthalein complexone assay (OCP), as previously described [40]. In brief, the cells were seeded in 48-well plates (15,000 cells/well) and then treated for 14 days in a mineralisation medium (Min) (Promocell, Heidelberg, Germany). The cells were then washed several times with phosphate buffer saline (PBS) and decalcified with 300 μL 0.6 N HCl/well for 2 h at room temperature on an oscillating stirrer. The calcium content of the HCl supernatant was colorimetrically determined using the o-cresolphthalein complexone assay, and absorbances were measured at 565 nm. The intracellular calcium content was normalised to the protein content using the Peterson assay [41]. Optical densities were measured at 750 nm.

### 4.6. Alkaline Phosphatase Activity

The alkaline phosphatase (ALP) activity was measured using the BioVision alkaline phosphatase activity colorimetric assay Kit (CliniSciences, Nanterre, France). The cells were seeded in 6-well plates (182,000 cells/well). Seven days post-treatment, the cells were washed with PBS and an ALP assay buffer was added to each well. In a 96-well plate, 10 μL of each sample to be tested was incubated with 70 μL ALP assay buffer and 50 μL 5 mM with pNPP solution. In parallel, a standard curve was prepared with a serial dilution of pNPP added to the ALP enzyme solution. After incubation (1 h, room temperature, protected from light), the reaction was stopped by adding 20 μL stop solution, and the absorbance was measured at 405 nm. The results were normalised to the protein content determined using the Pierce ™ BCA Protein Assay Kit (Thermofisher Scientific, New York, NY, USA). The reaction was incubated for 15 min at 56 °C. The absorbance was then measured at 565 nm.

### 4.7. Real-Time PCR Analysis

To study the expression of the genes involved in osteoblast differentiation and mineralisation, q-PCR was carried out at different times post-treatment for the HOb cells. The cells were seeded in 6-well plates (182,000 cells/well). After 2, 4, 6, 8, 10, or 12 days of treatment, RNA was isolated from the cells using the Trizol method (Thermofisher Scientific, New York, NY, USA). First-strand cDNA was generated from 1 μg total RNA by using the “maxima First Stand cDNA Synthesis” kit (Thermofisher, New York, NY, USA). cDNAs amplification was carried out by qPCR using Power SybrGreen (Applied Biosystems^®^, Thermofisher, New York, NY, USA) with a 7500 Fast Real-Time PCR system (Applied Biosystems, Thermofisher, New York, NY, USA). After 40 amplification cycles, the cycle threshold (Ct) was quantified according to the formula 2-ΔΔCt for each gene studied and normalised to glyceraldehyde-3-phosphate gene expression (GAPDH, Table 1), a housekeeping gene.

### 4.8. Transcriptomic Analysis

Gene expression profiling was performed in HOb cells treated with either Min, Min + 30 µM DP2, or Min + 100 ng/mL BMP-2 for 2 days. In brief, the Agilent RNA Spike-In mix was added to 100 ng of total RNA and reverse-transcribed to cDNA, followed by in vitro transcription and cyanine 3 labelling. The samples were then purified and hybridised on the SurePrint G3 Human Gene Expression 8 × 60 K v2 Microarray (Agilent Technologies, Santa Clara, CA, USA) for 17 h at 65 °C. After washing, the processed slides were scanned with the Agilent G2565BA microarray scanner (Agilent Technologies, Santa Clara, CA, USA). Raw data were extracted using Feature Extraction (version 10.7.1.1; Agilent Technologies). Next, quantile normalisation and subsequent data processing were performed using the Genespring software (version 12.0; Agilent Technologies, Santa Clara, CA, USA).

### 4.9. Western Blot

Western blotting was performed at various times post-HOb cell treatment. Two wells per condition were seeded at 100,000 cells/well in 6-well plates. After either 15 min, 30 min, or 2 h of treatment, the cells were washed with cold PBS, lysed in the RIPA buffer (HEPES, NaCl, EDTA, SDS, sodium deoxycholate, triton-X100, sodium fluoride, sodium orthovanadate, NaPP sodium pyrophosphate, H_2_O) and sonicated. After centrifugation, the supernatant containing the proteins was collected and a protein assay was performed using the Pierce ™ BCA Protein Assay Kit (Thermofisher Scientific, New York, NY, USA) as described above. For each experimental condition, 25 μg of proteins was separated by electrophoresis for 2 h at 90 V, and transferred onto a nitrocellulose membrane for 1 h at 100 V. After blocking with 5% BSA in TBS-Tween (TBST), the membranes were incubated overnight at 4 °C with various diluted primary antibodies (Table 2). The GAPDH protein was used as an internal control. The membranes were washed with TBST and then incubated for 1 h at room temperature with a secondary anti-rabbit IgG antibody labelled with HRP (goat anti-rabbit HRP). The protein bands were detected with the Amersham™ ECL™ Prime Chemiluminescence Solution (Bio-Rad, Hercules, CA, USA) and revealed using the ChemiDoc MP Imaging System (Bio-Rad, Hercules, CA, USA). Band analysis was performed using ImageJ software (version 1.52a, NIH, Bethesda, USA).

### 4.10. Fluorescence Microscopy

HOb cells were seeded at 100,000 cells on glass coverslips. A treatment kinetic study with fluorescent DP2 (λ ex: 455 nm, λ em: 514 nm) was performed: 15 min, 30 min, 2 h (h), 4 h, 6 h, 8 h, 10 h, 24 h, on D2, D4, D6, D8, D10, D12, and D14. After treatment, the cells were fixed with 4% paraformaldehyde (PFA, SIGMA, St. louis, MO, USA), cell membranes were labelled using a PKH26 Red fluorescent kit (SIGMA, St. louis, MO, USA), and nuclear staining was performed by incubating the cells with Hoechst 33,342 (1/5000; Thermofisher scientific, New York, NY, USA). The coverslips were then mounted in a mounting medium (DPX; SIGMA, St. louis, MO, USA) and observed using a confocal microscope (LSM780; Carl Zeiss, Oberkochen, Germany), then analysed using the Imaris x64 software (9.5.1, Oxford instruments, Abingdon-On-Thames, UK).

### 4.11. Statistical Analysis

The results are expressed as the mean ± the standard error of the mean (SEM). Statistical procedures were performed using GraphPad Prism 9.00 software (GraphPad Software, San Diego, CA, USA). Before any analysis, the Gaussian distribution of the studied parameters was checked (normality test). Based on the result of the normality test, statistical differences between the groups were measured using the corresponding test: the Mann–Whitney test, the Wilcoxon signed-rank test, one-way ANOVA followed by the Kruskal–Wallis comparison test, or two-way ANOVA followed by the Tukey multiple comparison test. A value of *p* < 0.05 was considered significant.

## 5. Conclusions

Currently, few therapeutics are used to promote bone formation following bone degeneration or fracture. In the present study, we reported on a new osteogenic molecule and determined the underlying mechanism of action. Our results show that DP2 induces HOb cell mineralisation in a similar manner to BMP-2. However, DP2 seems to enhance osteoblast differentiation earlier than BMP-2. These results should be confirmed in an in vivo model of bone reconstruction. As such, DP2 should be considered as a potential therapeutical molecule in delayed bone fracture repair. DP2 could be used for orthopaedic treatments, such as osteotomy and pseudarthrosis. Interestingly, DP2 can be combined with autographs for patients with comorbidities such as diabetics, smokers, and anaemia.

## Figures and Tables

**Table 1:** Primer sequences used to quantify gene expression in a RT-qPCR assay.

Gene	Forward	Reverse
hGAPDH	5′-TCGGAGTCAACGGATTTGG-3′	5′-GCAACAATATCCACTTTACCAGAGTTAA-3′
hRunx2	5′-GGCCCACAAATCTCAGATCGTT-3′	5′-CACTGGCGCTGCAACAAGAC-3
hOCN	5′-TGCAGAGTCCAGCAAAGGTGCA-3′	5′-ATAGGCCTCCTGAAAGCCGATGT-3′
hOPN	5′-TCACAGCCATGAAGATATGCTGG-3′	5′-TACAGGGAGTTTCCATGAAGCCAC-3′

**Table 2:** Antibodies used in Western blotting.

Antibody	Molecular Weight	Catalogue Number	Dilution	Ref.
Rabbit anti-protein kinase c (PKC)	80 kDa	2058	1/1000	Cell signalling (Leiden, The Netherlands)
Rabbit anti-phospho PKC	80, 82 kDa	9374		
Rabbit anti-glyceraldehyde-3-phosphate dehydrogenase (GAPDH)	37 kDa	2118		
Goat anti-rabbit HRP		7074		

## Data Availability

The data presented in this study are available on request from the corresponding author. The authors can confirm that all relevant data are included in this article.
